# EHPnet: International Maritime Organization

**Published:** 2006-04

**Authors:** Erin E. Dooley

In 1948 an international conference in Geneva adopted a convention formally establishing what is today called the International Maritime Organization (IMO). The IMO initially worked mainly to ensure maritime safety, but after a 1967 spill of 100,000 tons of crude oil off the southern coast of England, the organization began focusing attention on alleviating the environmental impacts of the shipping industry. Today the IMO has devoted a section of its website at **http://www.imo.org/home.asp** to these environmental programs.

The Marine Environment section, accessible through the menu at the top of the IMO homepage, provides an overview of how the IMO works to regulate and prevent marine pollution by ships, with links to in-depth information on the applicable international treaties. The first such treaty is the International Convention for the Prevention of Pollution from Ships (MARPOL 73/78), which was adopted in 1973 and modified in 1978. This treaty governs accidental and operational oil pollution as well as pollution by chemicals, packaged goods, sewage, garbage, and air pollution. The 1990 International Convention on Oil Pollution Preparedness, Response and Co-operation calls on parties to establish measures for reporting and handling oil pollution incidents. The IMO also serves as secretariat for the Convention on the Prevention of Marine Pollution by Dumping of Wastes and Other Matter, which was adopted in 1972.

A list of links on the Marine Environment page leads to other topics of interest. The Ship Recycling page has a detailed overview of the IMO’s moves to govern the disassembly and recycling of ships. Although the organization adopted recommended guidelines on ship recycling in 2003, the IMO’s senior technical body agreed in 2005 to develop legally binding regulations for the design, construction, operation, and preparation of ships to enable safer and more environmentally sound recycling, along with rules for enforcing the instrument. The Ship Recycling page also has links to an IMO article on ship recycling, to pages on the current guidelines for ship recycling, and to the website for the joint IMO/International Labour Organization/Basel Convention Working Group on Ship Scrapping.

The Prevention of Pollution section has links to pages on specific forms of pollution covered by the conventions that the IMO is responsible for (such as oil pollution, chemical pollution, sewage, and air pollution). These pages provide information on the specific protocols that govern each area, background on the pollution source and the problems it can cause, and details about how the treaties came about. Within this section there is also a page about shipboard pollution prevention equipment required under MARPOL 73/78.

The Ballast Water Management section includes information on the International Convention for the Control and Management of Ships’ Ballast Water and Sediments, which was adopted in February 2004, as well as information in seven languages on other IMO guidelines covering this subject. There is also an external link to the site for the Global Ballast Water Management Programme, a partnership between the IMO, the UN Development Programme, and the Global Environment Facility that seeks to help developing countries understand the problem of ballast contamination and prepare to implement the convention.

